# Application of robotics In the clinical laboratory

**DOI:** 10.1155/S1463924689000131

**Published:** 1989

**Authors:** R. A. Bunce, P. M. G. Broughton, D. M. Browning, J. E. C. Gibbons, L. J. Kricka

**Affiliations:** 1 Department of Clinical Chemistry Wolfson Research Laboratories Queen Elizabeth Medical Centre Birmingham B15 2TH UK; 2 Department of Clinical Chemistry University of Birmingham Birmingham B15 2TH UK; 3 Department of Pathology and Laboratory Medicine William Pepper Laboratories Hospital of the University of Pennsylvania 3400 Spruce Street Philadelphia PA 19104-4283 USA

## Abstract

The basic types of robot are explained, and the performances and costs of some commercial examples are given. The potential advantages and problems of introducing robots into clinical laboratories are identified and the specifcation of a suitable robot is developed. None of the commercially available robots meets all aspects of the specificalion, and currently the purchase of a robot is considered premature for most clinical laboratories.


Journal of Automatic Chemistry Vol. 11, No. 2 (March–April 1989), pp. 64–69

### Application of robotics in the clinical laboratory

**R. A. Bunce,† P. M. G. Broughton, D. M. Browning, J. E. C. Gibbons**

*Department of Clinical Chemistry, Wolfson Research Laboratories, Queen Elizabeth Medical Centre, Birmingham B15 2TH, UK*

**and L. J. Kricka‡**

*Department of Clinical Chemistry, University of Birmingham, Birmingham B15 2TH, UK*

*The basic types of robot are explained, and the performances and costs of some commercial examples are given. The potential advantages and problems of introducing robots into clinical laboratories are identified and the specification of a suitable robot is developed. None of the commercially available robots meets all aspects of the specification, and currently the purchase of a robot is considered premature for most clinical laboratories.*

### Introduction

A robot can be defined as an apparatus that can perform manipulations in three dimensions and can be programmed so that these manipulations are repeated or varied without human intervention over an extended period of time.

Robots are now being used widely in industry for assembly, machining, finishing and automatic chemical analysis. They have also been advocated for use in the clinical laboratory to automate repetitive analytical procedures [1]. Here they could have a role where the alternative of a dedicated analyser is either not available or is unsuitable because, for example, medium-sized batches of different types of test need to be assayed. Currently there are a number of robots available commercially which may be considered for clinical laboratory purposes (table 1). Some of these have been used to automate a range of clinical analyses, e.g. enzyme immunoassay, drug analysis, UV–visible spectrophotometric assay and bioassay [2–8]. Dedicated robotic arms have also been used for specimen/reagent transfer in automatic clinical chemistry analysers such as the Instrumentation Laboratories Monarch and the Roche Cobas-Fara, and in sample preparation units such as the Tecan RSP 510 Sampler and Kemble Star 700 instrument.

This paper briefly reviews the non-dedicated types of robot currently available, discusses the features which are of potential value for clinical laboratories, summarizes the important factors to be considered in buying a robot and proposes a specification for a robot suitable for use in clinical laboratories.

### Main types of robot available

Robots may be static (e.g. floor, bench or ceiling mounted) or mobile on a tracked system (table 2).

The mechanical functions of a robot can be likened to those of the human body. The basic element is the trunk, to which is attached one or occasionally two arms which in some instruments have elbow joints. A variety of accessories can be attached to the end of the arm, such as a hand, fingers or special devices for pipetting, etc. The range of these accessories varies between manufacturers, but they are not necessarily interchangeable between different robots. The accessories available should therefore be considered before choosing a robot.

Robots can be classified into five general types:

(*a*) *Revolute.* A robot consisting of rotational members arranged in a human hand, arm, shoulder and trunk configuration (e.g. Perkin-Elmer Masterlab Robot) [9].

(*b*) *Cylindrical.* A robot consisting of a central vertical sliding and rotating unit into which slides a horizontal beam (e.g. Zymark Zymate) [10].

(*c*) *Selectively compliant articulated robot arm (SCARA).* A robot consisting of a central vertical sliding unit to which is attached two horizontally rotating links (e.g. Universal Machine Intelligence RTX Robot).

(*d*) *Cartesian.* A robot consisting of a vertical sliding unit which is mounted on an $x$-axis slideway carried on a $y$-axis horizontal sliding beam (e.g. experimental laboratory robot by the Laboratory of the Government Chemist.

(*e*) *Polar.* A robot consisting of an extending horizontal beam, pivoted vertically at one end, mounted on a rotating unit (e.g. GEC Robot Systems RAMP 2000).

Numerous examples of each type are available commercially and some are listed in table 1.

### Characteristics of potential importance in using a robot in a clinical laboratory

The two main potential advantages of using a robot in a clinical laboratory are safety and unattended and extended operation.

### *Safety*

A robot may safely handle hazardous reagents (e.g. carcinogens, radioactive compounds and infectious

†*To whom correspondence should be addressed.*
‡*Present address: Department of Pathology and Laboratory Medicine, William Pepper Laboratories, Hospital of the University of Pennsylvania, 3400 Spruce Street, Philadelphia, PA 19104–4283, USA.*

0142–0453/89 $3.00 © 1989 Taylor & Francis Ltd.

*Table 1. Robots which may be considered for use in clinical laboratories.*

| Supplier | Robot make model type | Cost of robot and controller | Country of origin | Reach (mm) | Payload (kg) | Speed (mm/s and °/s) | Repeat-ability (± mm) | Degrees of freedom | Laboratory accessories available |
|---|---|---|---|---|---|---|---|---|---|
| Zymark Ltd. | Zymark<br>Zymate II<br>Cylindrical | £16 700 | USA | 660 | 1·4 | 640<br>60<br>Variable | <1·0 | 4 | Yes |
| Unimation Inc. | Unimation<br>Puma 260 MK3<br>Robot arm | £31 000 | USA/UK | 406 | 0·9 | 1000<br>—<br>Variable | 0·05 | 6 | No |
| Unimation Inc. | Unimation<br>Puma 552 MK3<br>Robot arm | £28 000 | UK | 1000 | 4·0 | 500<br>—<br>Variable | 0·1 | 5 | No |
| Unimation Inc. | Unimation<br>Puma 562 MK3<br>Robot arm | £34 000 | UK | 1000 | 4·0 | 500<br>—<br>Variable | 0·1 | 6 | No |
| Perkin-Elmer Ltd. Masterlab | Mitsubishi<br>RM-501<br>Robot arm | £20 000 | USA/Japan | 660 | 1·2 | 400<br>—<br>Variable | 0·5 | 5 | Yes |
| Peerless Systems Ltd. | Peerless<br>Track mounted<br>Cylindrical | £11 000 | UK | 500 | 2·0 | 500<br>120<br>Variable | 0·1 | 6 | Yes |
| Hudson Robotics Inc. | CRS Plus<br>SRS–MIA<br>Robot arm | $19 300 | USA/Canada | 559 | 2·0 | 1016<br>60–180<br>Variable | 0·1 | 5 | Yes |
| Hudson Robotics Inc. | Mitsubishi<br>RVM1<br>Robot arm | $15 300 | USA/Japan | 483 | 1·2 | 1000<br>—<br>Variable | 0·3 | 5 | Yes |
| Universal Machine Intelligence Ltd. | UMI Ltd<br>RTX<br>Modif.: Scara | £7480 | UK | 685 | 4 | 1100<br>43<br>Variable | 0·5 | 6 | Yes |

Specifications provided by manufacturers, December 1988.

*Table 2. Mobile robots.*

| Supplier | Additional cost of mobility | Country of origin | Type of robot and degree of mobility |
|---|---|---|---|
| Perkin Elmer Ltd. | £3750 | USA/Japan | Masterlab Robot mounted on a 3-m track |
| Hudson Robotics Inc. | $8900 | USA/Japan | Mitsubishi RMV1 Robot mounted on a track 6–50 ft long |
| Hudson Robotics Inc. | $7625 | USA/Canada | CRS PLUS SRS–MIA Robot mounted on a 6–50-ft track |
| Zymark Ltd. | £1500† | USA/UK | Zymate II Robot mounted on a track |
| Peerless Systems Ltd. | £1500† | UK | Peerless Robot mounted on a track |

† Price per metre of track.
Information supplied by manufacturers, December 1988.

specimens) [11–13]. This is particularly important in view of the dangers of handling biological material contaminated with infectious agents such as HIV-III or hepatitis virus. A robot could be used to analyze clinical specimens in a controlled environment and thus minimize human contact with samples, or reagents (environmental chamber, Hudson Robotics). However, a robot used for such a purpose might need to be decontaminated before it could be safely accessed by a human operator (e.g. for servicing, repair or reloading). Little consideration seems to have been given to methods of decontaminating a robot or disposing of hazardous waste handled by a robot.

### *Unattended and extended operation*

A robot is capable of unattended operation so that one or more assays could run without human intervention on a 24-h basis or longer. This would free laboratory staff for other tasks and thus increase productivity. A robot would be particularly suited to the automation of a mediumsized workload of complex tests which would be unsuitable for either a dedicated instrument or a manual method [14]. In the short term, robot technology is unlikely to replace current automated equipment but can form an important physical link between analytical devices.

The introduction of robots could have far reaching implications in the clinical laboratory by influencing staffing levels, costs, laboratory organization and employee education. The design of analytical instruments will need to be compatible with both robots and computers, and laboratory design will be influenced by the new technology.

The wider consequences of robotics in the clinical laboratory are largely speculative. Comparisons can be made with previous innovations in laboratory automation such as the introduction of AutoAnalyzers, computers and, more recently, selective discrete analyzers. Judicious use of the latter is beginning to reduce staff numbers and costs. Clearly, robotics are another important step towards complete automation, but are unlikely to result in immediate cost reductions. It is more likely that the introduction of robots will follow the industrial pattern and reduce repetitive manual jobs (e.g. pipetting, weighing) and increase output.

Present-day robots are neither self-repairing nor able to make intelligent decisions. They are only as skilful as the programming and as reliable as their design, construction and component quality. Programmers, engineers and chemists will be needed to tailor systems to specific requirements and to direct the day-to-day running of robots.

At present both manufacturers and users are not paying enough attention to robot safety. A common misconception is that it is safe to allow a robot arm to strike personnel provided that the force is low and that the robot would automatically stop. Robots may operate discontinuously and this can lead to a false sense of security. In common with any other mechanism, they should be properly guarded in accordance with the appropriate standards.

### Provisional specification for a clinical laboratory robot

This specification is intended to serve as a guide for both manufacturers and prospective users.

*Type of robot*

*Definition.* See above.

*Specification.* Revolute and cylindrical types appear to be the most suitable.

*Rationale.* SCARA type robots do not normally cover 360° of horizontal freedom. This limits the number of analytical modules which can be served within the reach of a given robot. Cartesian robots need a superstructure encompassing the analytical modules. This superstructure would be specific for a particular task and thus unsuitable for general analytical applications. The polar type is less versatile than either the cylindrical or revolute types of robot.

The revolute is the most versatile robot, but the construction is more complex and the fundamental programming very sophisticated, so costs are higher. A distinction needs to be made between fundamental programming written by manufacturers of the robot and application programming usually written by the user. The cylindrical type is reasonably versatile involving less demanding engineering and fundamental programming.

Practical experience with the revolute and cylindrical types is necessary to decide which is the most suitable for clinical laboratories.

*Working envelope*

*Definition.* The surface generated by the flange of the hand of the robot or robot system at extremes of reach.

*Specification.* Either a cylinder (360° of reach) in the case of cylindrical robots or a half sphere in the case of revolute robots, with a radius equal to the reach. (Note: a robot with a spherical envelope has no vertical rise of the hand at extreme reach and care needs to be exercised when comparing the two types.)

*Rationale.* A circular layout is required so that the minimum size of robot can serve the maximum number of peripheral instruments in the minimum time. A rectangular layout may be more easily accommodated on existing laboratory benches but it gives an inherently slow access time to instruments. A spherical layout could also be considered, in which case the peripheral instruments would be at different heights above the bench surface.

*Reach*

*Definition.* The maximum distance from the centre line of the robot to the fixing flange of the hand (see also *Working envelope*).

*Specification.* 0·5–1·0 m.

*Rationale.* This will give an area 1–2 m in diameter that is accessible to the robot and within which all peripheral equipment should fit. Such a free working area would require a room or space of not less than 3–5 m square in order to include access by personnel.

*Handling capacity*

*Definition.* The mass which can be held in the robot's hand that does not unduly degrade the speed, acceleration and deceleration of the robot. This is not necessarily equal to the static or dynamic forces produced by the robot.

*Specification.* 2 kg, excluding the mass of the robot's hand. Speed, acceleration and deceleration should not be degraded by more than 10%.

*Rationale.* 2 kg is the mass of a specimen turntable of a typical analyzer (e.g. Hitachi 737) fully loaded with 60 specimen tubes each containing 10 ml of whole blood.

*Degrees of freedom*

*Definition.* The number of rotational axes and sliding members associated with the main elements of the robotic arm.

*Specification.* 5 or 6.

*Rationale.* Four degrees of freedom are essential, i.e. $X$, $Y$ and $Z$ movements and also hand rotation (roll) for tipping from one vessel to another. In addition, the use of screw-cap containers, for example, would require a further axis at the wrist/hand—one more degree of freedom than in a human. This clearly adds to the versatility and its use needs further investigation.

*Repeatability*

*Definition.* The repeatability of the positioning of a point on or near the hand of the robot anywhere within the working envelope under specified combinations of speed, acceleration/retardation and load.

*Specification.* ±0·5 mm.

*Rationale.* This is the repeatability judged necessary to locate and position various analytical vessels or carriers such as test tubes, specimen cups, microtitre plates and turntables. Only practical experience will determine if this figure is justified.

*Speed*

*Definition.* The uniform linear or rotational speed of a robotic hand under specified loading conditions.

*Specification.* Variable up to 1000 mm/s at the hand.

*Rationale.* The slowest speed acceptable for a particular task is determined by comparison with a human operator. Faster speeds usually involve higher accelerations and the ability to handle liquids precisely is dependent on the shock forces imposed by these accelerations. Hence, the upper speed is determined by the acceptable precision of liquid handling. Further, it is known that the speed of human hands can easily cause unacceptable imprecision. The upper speed limit has been chosen with this in mind. This aspect needs practical assessment.

*Robotic hands*

*Definition.* Robotic hands are devices which can be fitted to the distal end of the arm of a robot either manually or automatically; they are designed to grasp objects, inject liquids, incorporate analytical probes, specimen recognition devices, etc.; such features can be incorporated singularly or in combination in each hand.

*Specification.* A series of hands have been identified:

1. Basic hand for grasping test-tube type vessels from 30 × 8 mm o.d. to 140 × 16 mm o.d. This would also be used for grasping probes used for measuring pH, etc.

2. A hand for grasping microtitre plates (which typically measure 130 × 90 × 16 mm).

3. Hands designed to grasp or incorporate 'manually operated' pipettes.

4. Hands designed to grasp or incorporate automatic pipettes or their tips.

5. Hands designed to insert and remove both push-on and screw-on types of stoppers on vessels.

6. Hands designed to grasp turntables.

These various hands may be adaptations of the basic hand by, for example, fitting appropriate fingers. The method of gripping may be based on mechanical fingers, vacuum suckers, electromagnets, etc. Practical experience will identify additional types of hand but in each instance the hand must be capable of being fitted automatically to the robot.

*Rationale.* The list of hand requirements is based on established clinical laboratory equipment and techniques.

*Control unit*

*Definition.* The control unit of the robot is that unit, usually involving a computer, which interprets the programming instructions of the human operator and coordinates and directs the various electromechanical elements of the robot and ancillary equipment to do predetermined tasks. In addition, the control unit monitors these actions, acts on this information and, if necessary, reports to the operator.

*Specification.* 1. The controller must be simple to use and involve no specialized programming knowledge, and should be outside the cover (see *Robot safety*).

2. The controller must be capable of coordinating the various mechanical elements of the robot so that, for example, the hand can be made to move in simple geometric patterns such as straight lines without the need for the operator to programme individual elements.

3. The controller should allow the operator to instruct the robot using the following methods:

(a) By the 'inch control' of individual motions.

(b) By the specification of $X$, $Y$ and $Z$ coordinates for a point on or near the hand, e.g. pipette tip. The starting point of the $X$, $Y$ and $Z$ coordinates is defined anywhere within the working envelope of the robot and at any angle; for example, the $Z$ coordinate is usually vertical, but not necessarily so.

(c) By using an auxiliary, hand-held, remote control unit.

(d) By the definition of a matrix system such as the coordinates of two extreme, diagonally opposed wells of a microtitre plate. In that case the controller would determine the position of all the wells automatically.

(e) By manually moving the robotic hand.

4. The controller must be easily interfaced to a laboratory computer and analytical modules. It must also be capable of stand-alone operation with limited data procesing.

5. The controller must automatically smooth out sudden changes of direction.

6. The speed and acceleration of the robot must be variable and be programmable.

7. The emergency stop procedure must be fail-safe and not software dependent.

*Rationale.* The specification is based on our experience of advanced laboratory automation and computing. It is important that the robot has an advanced control system which is reliable and well established. It would be very difficult for laboratory staff to contribute to fundamental software development.

*Training courses*

*Definition.* Courses run by the manufacturer of robots to enable users to operate a particular robot effectively.

*Specification.* The training course should cover the following aspects and should be tailored to suit individual needs and disciplines:

1. Programming.
2. Interfacing.
3. Fault finding.
4. Safety.
5. Applications.

It will be necessary to have introductory courses, which should assume no previous knowledge of robotics, and in addition advanced courses. The manufacturer should provide all information necessary for the effective and safe use of the robot.

*Rationale.* A robot system is a complex and versatile piece of automation embodying computer technology and precision electromechanical engineering. It is essential that manufacturers should provide proper information to enable robots to be operated effectively and safely. This must include a training course so that the novice can gain hands-on experience under the guidance of experts.

*Robot installation*

*Definition.* A robot installation is the total robotic system including services such as water supply and drainage, electrical power supply, working area, mounting requirements for robot and any associated equipment and all safety features such as interlocked guarding and doors.

*Specification.* The robot must be properly installed with due regard to the following:

1. It must be securely mounted to a stable structure such as a bench or floor to prevent vibrational movement or accidental movement by personnel.

2. Sufficient area must be allocated for the robot, interlocked cover and auxiliary equipment such as power supply or control units. This must also include reasonable access and room for servicing.

3. Services such as water supply, drains and electrical power must be properly installed.

4. Safety aspects of the whole installation must comply with appropriate standards.

*Rationale.* A robot, like any other piece of clinical laboratory automation, must be properly installed and comply with the relevant standards. In addition, installing a robot is certain to raise new problems and these can only be addressed and studied practically.

*Robot safety*

*Definition.*The prevention of mechanical injury, electrical shock, infection or poisoning to laboratory staff or bystanders either directly or indirectly associated with the robot system.

*Specification.* The robotic system shall comply with all relevant safety regulations. In particular:

1. The robotic system shall have a totally enclosed transparent cover covering the whole of the robot and peripheral instruments but excluding the controller, which must be located outside the cover.

2. There must be a means of access through the cover to the robot. This must be interlocked so that access is permitted only when conditions inside the cover are safe.

3. The cover must be constructed so that it is not damaged by impact from the robot.

4. Substances which give off hazardous vapours must not be used inside the cover in order to minimize the risk of corrosion, fire or explosion, unless the covered area has an extractor.

5. Precautions must be taken to prevent high humidity inside the cover in order to safeguard electrical circuits.

6. Facilities must be provided to deal with spillages inside the cover without the need for human access.

7. The robot, peripheral instruments and control unit must be designed to prevent ingress of toxic substances. The texture, colour and shape of all surfaces must allow easy decontamination.

8. The system shall be designed to be fail-safe, and this must include special precautions in the event of electrical mains, water drainage or compressed air failure, etc.

9. The control system system must be arranged to give visual/audio warning of malfunction. Ideally, this should include a telecommunication link to a remote operator in the case of unattended operation.

*Rationale.* Robots are new to the clinical laboratory and are expected to present special problems involving safety. Safety aspects must be thoroughly investigated practically before toxic substances are handled by clinical laboratory robots.

*Servicing and maintenance*

*Definition.* The service provided by the manufacturer to enable the robotic system to run efficiently.

*Specification.* The manufacturer shall provide a 24-h repair service and also an optional maintenance contract. These shall be guraranteed to continue for the expected life of the robot which should be not less than 5 years.

*Rationale.* For robotic systems to be cost effective they must have the capability of working continuously during the day and night. Hence, robotic systems require similar backup facilities to those conventional instruments which are designed for 24-h use.

*Peripheral instruments*

Robots will be used with a variety of peripheral instruments, most of which are not available from the robot manufacturer. In selecting a robot, the type of peripheral instruments to be used needs to be considered, for example their size, arrangement within the working envelope and the dexterity needed by the robot for the robot to operate them.

*Definition.* Analytical instruments such as photometers, pipettes and incubators which are to be used with the robot system.

*Specification.* All peripheral instruments must be capable of non-dedicated operation, that is, they must be robot and human compatible.

*Rationale.* The essence of a robotic system is its flexibility. Hence, peripheral instruments must be capable of being used manually both inside and outside the robotic system either for a particular application or in the case of robot failure.

### Discussion

The specifications outlined here involve many compromises. For example, additional handling capacity or reach would increase the working envelope and cost, and extra speed is likely to influence the precision adversely. It is therefore important for the user to consider all these factors before choosing a robot.

It is still not clear whether the introduction of robots into the clinical laboratory will be beneficial. Likewise, it is unclear which analyses will benefit from robotics. A successful robot will require a very flexible software control system and a range of hands in order to meet the diverse needs of clinical laboratory analysis.

Automation based on inexpensive teaching robots is not to be recommended because they are not sufficiently robust. Laboratory robots are relatively new and there is very little electronic/mechanical compatibility between the various suppliers of robotic elements and laboratory instrumentation. Hence, it is recommended that, at present, laboratory users should purchase complete laboratory robotic systems from one supplier. However, few of the currently available robots have the required flexibility and range of accessories and none meet the draft specifications proposed in this paper. Therefore, currently the purchase of a laboratory robot would be premature for most clinical laboratories.

### Acknowledgement

The financial support of the Department of Health is gratefully acknowledged.

### Appendix

*Robot suppliers*

Hudson Robotics Inc., 120 Morris Avenue, Springfield, NJ 07081, USA.

Peerless Systems Ltd, 332a Mayoral Way, Team Valley, Gateshead, NE11 0RT, UK

Perkin-Elmer Ltd, Post Office Lane, Beaconsfield, Buckinghamshire HP9 1QA, UK

Unimation Inc., Unit G, Stafford Park 18, Telford, Shropshire TF3 3AX, UK

Universal Machine Intelligence Ltd, 9–15 St James Road, Surbiton, Surrey KT6 4QN, UK

Zymark Ltd, The Genesis Centre, Science Park South, Birchwood, Warrington WS3 7BH, UK

### References

1. Roberts, L. B., *Medical Laboratory World*, January (1986), 21.
2. Hawk, G. L. (Ed.), *Advances in Laboratory Automation Robotics* (Zymark Corp., Hopkinton, MA, 1984 onwards annually).
3. Zymark Corporation Inc., *Technical Brief: Automated ELISA assays* (Zymark Corp., Hopkinton, MA, 1985).
4. Williams, F. J. and McGrattan, P. A., *International Instrumentation-Research*, March (1986), 24.
5. Castellani, W. J., Van Lente, F. and Choy, D., *Clinical Chemistry*, **32**, (1986), 1672.
6. Luders, R. C. and Brunner, L. A., *Journal of Chromatographic Science*, **25** (1987), 192.
7. Johnston, G., *Laboratory Equipment Digest*, July (1987), 52.
8. Hurst, W. J. and Mortimer, J. W., in *Laboratory Robotics* (VCH, Weinheim, 1987).
9. Barrett, P., *International Instrumentation-Research*, September (1985), 48.
10. Stafford, J. E. H., *Laboratory Practice*, March (1985), 19.
11. Friedman, L. I. and Stevens, M. L., *Vox Sang.*, **51**, Suppl. 1 (1986), 57.
12. Brodack, J. W., Kilbourn, M. R. and Welch, M. J., *Journal of Nuclear Medicine*, **27** (1986), 714.
13. Brennan, J. E., Severns, L. M., Kline, L. M. and Epley, K. M., *Vox Sang.*, **54** (1988), 115.
14. Roberts, L. B., *Journal of Automatic Chemistry*, **10** (1988), 4.

